# Alignment of the Cervix with the Vagina in Uterine Retroversion: A Possible Risk Factor in Uterine Prolapse

**DOI:** 10.3390/diagnostics12061428

**Published:** 2022-06-09

**Authors:** Alan H. Appelbaum, Mehran Tirandaz, Giuseppe Ricci, Roberto Levi D’Ancona

**Affiliations:** 1 Memphis Veterans Affairs Medical Center, 1030 Jefferson Avenue, Memphis, TN 38104, USA; mehran.tirandaz@va.gov; 2 University of Tennessee Health Science Center, Memphis, TN 38163, USA; 3 Institute for Maternal and Child Health, IRCCS Burlo Garofolo, 34137 Trieste, Italy; giuseppe.ricci@burlo.trieste.it; 4 Department of Medicine, Surgery and Health Sciences, University of Trieste, 34127 Trieste, Italy; 5 Southern Illinois University School of Medicine, Springfield, IL 62702, USA; rlevi-dancona52@siumed.edu

**Keywords:** pelvic anatomy, pelvic floor, prolapse, retroversion, uterus, vagina

## Abstract

Multiple observational studies have found an association of uterine prolapse with uterine retroversion. Mechanisms proposed to explain this apparent association assume that the cervix of a retroverted uterus will usually insert at the apex of the vagina, with resultant alignment of the cervix with the vagina. The angle of the axis of the cervix with the axis of the vagina was measured by two readers on 323 sagittal pelvic MRI scans and sagittal reconstructions of pelvic CT scans performed for clinical purposes. One reader observed and recorded the anatomic relations of the uterus that differed by insertion site and version: 44 of 49 retroverted uteri (89.8%) inserted at the vaginal apex, and 13 of 274 anteverted uteri (4.7%) inserted at the vaginal apex. This difference was found to be statistically significant (*p* < 0.05) by the Chi square test. The urinary bladder, vaginal walls, and rectum were inferiorly related to anteriorly inserted anteverted uteri. Only the vaginal lumen and the rectum at a shallow oblique angle were inferiorly related to apically inserted retroverted uteri. Most retroverted uteri insert at the apex of the vagina. Apically inserted retroverted uteri appear to receive less support from adjacent structures than anteriorly inserted anteverted uteri.

## 1. Purpose

Two recent observational studies demonstrated a possible association of uterine prolapse with uterine retroversion [1,2]. There are similar studies showing this possible association dating back to the first half of the twentieth century [3,4]. We have found three proposed mechanisms to explain this apparent association. All three assume that the cervix of a retroverted uterus will usually insert at the apex of the vagina, with resultant alignment of the axis of the uterus with the axis of the vagina. One mechanism proposes that this alignment of the uterus with the vagina would allow the uterus to descend and ascend within the vagina with increases and decreases in intra-abdominal pressure. This piston-like action would create mechanical stress on the supporting structures of the uterus, leading to uterine prolapse and other pelvic floor dysfunctions [1,5]. A second mechanism proposes that the alignment of the uterus with the vagina in retroversion would ease passage of the uterus through the vagina when there is widening of the urogenital hiatus and other damage to the supporting structures of the uterus [6,7]. The third mechanism proposes that weakness of the uterosacral ligament allows the uterus to rotate posteriorly, allowing alignment of the uterus with the vagina. This rotation would twist and stress the transverse cervical ligaments, leading to prolapse [8].

Cross-sectional imaging has been successfully used to study the anatomy of the vagina, uterus, and related structures. For example, studies using MRI have been used to measure the lengths and diameters of the vagina and uterus [9,10,11], studies using MRI and ultrasound have been successfully used to measure the angle of the uterus with the vagina [12,13], and dynamic MRI has been used to evaluate the pelvic floor [14]. In the current study, we measured the angle of version of the uterus with the vagina in a series of female abdominopelvic and pelvic CT scans and pelvic MRI scans to attempt to determine if the assumed apical insertion with resultant alignment of the uterus with the vagina in retroversion is correct. We found no other studies that examined this question despite a thorough literature search. The measurements we obtained showed substantial, statistically significant evidence that the uterus aligns with the vagina in most cases of retroversion. This confirms that the anatomic alignment necessary for the proposed mechanisms of uterine prolapse does occur. Additional investigations to confirm or exclude that uterine retroversion is a causative factor for uterine prolapse and how this may occur are recommended.

## 2. Methods

After approval was obtained from the institutional review board of the Memphis Veterans Affairs Medical Center (IRBNet ID #753824; 2/25/19), measurements were conducted by two readers on multiple consecutive series of abdominopelvic and pelvic CT scans and pelvic MRI scans of female patients performed for clinical purposes at our institution. The enrollment periods were from January 2016 to November 2020 for CT scans and June 2007 to January 2019 for MRI scans. Scans were excluded from the study if the uterus was absent, if the uterus was prolapsed, or if the scan was not technically adequate to measure the angle between the uterus and vagina. The age of each woman in the study and the clinical indication for each scan were recorded. Both readers (A.A., M.T.) are senior staff radiologists at our institution.

The CT scans were performed on GE Revolution EVO or GE Revolution HD CT scanners. Sagittal reconstructions were performed on the workstations of the scanners. The MRI scans were performed on GE MR HDxt or GE HC MR DV scanners. Early in the period of the study, the MRI scans were routinely performed with distention of the vagina by 20 cc of water-soluble gel following the clinical protocol for that examination. Later in the study, gel distention of the vagina was omitted in most cases following a change in the clinical protocol.

**Figure 1:**
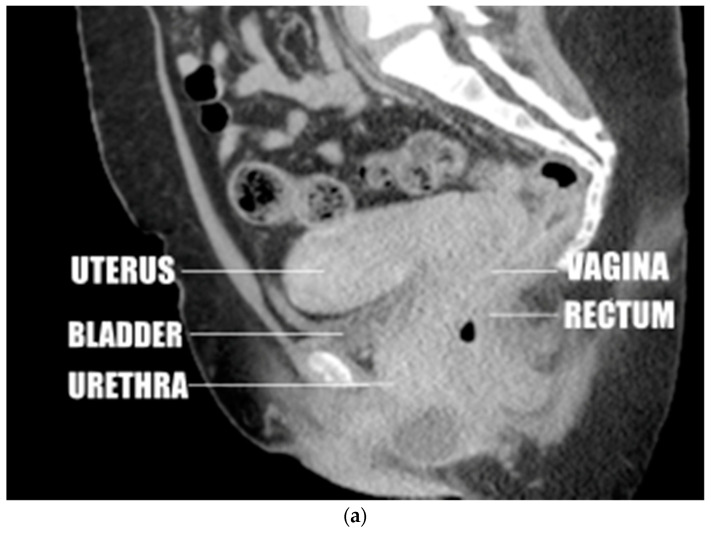

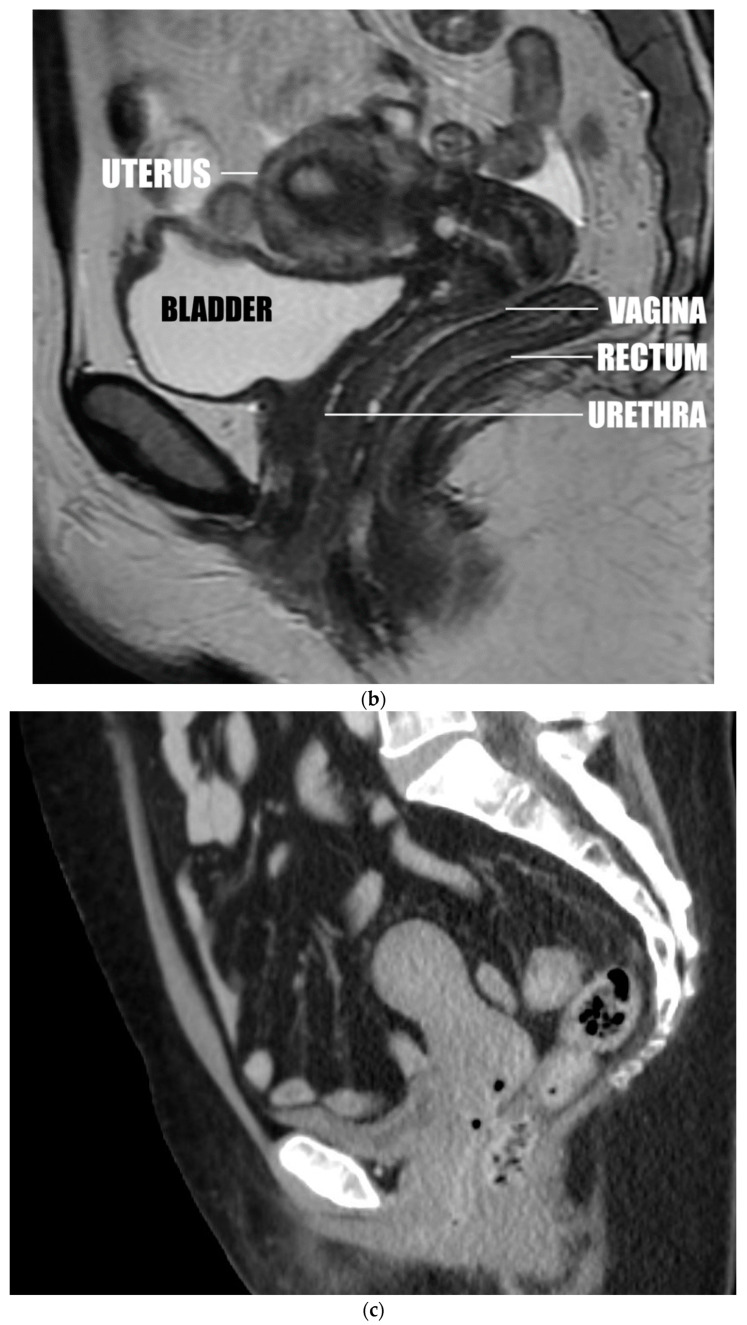

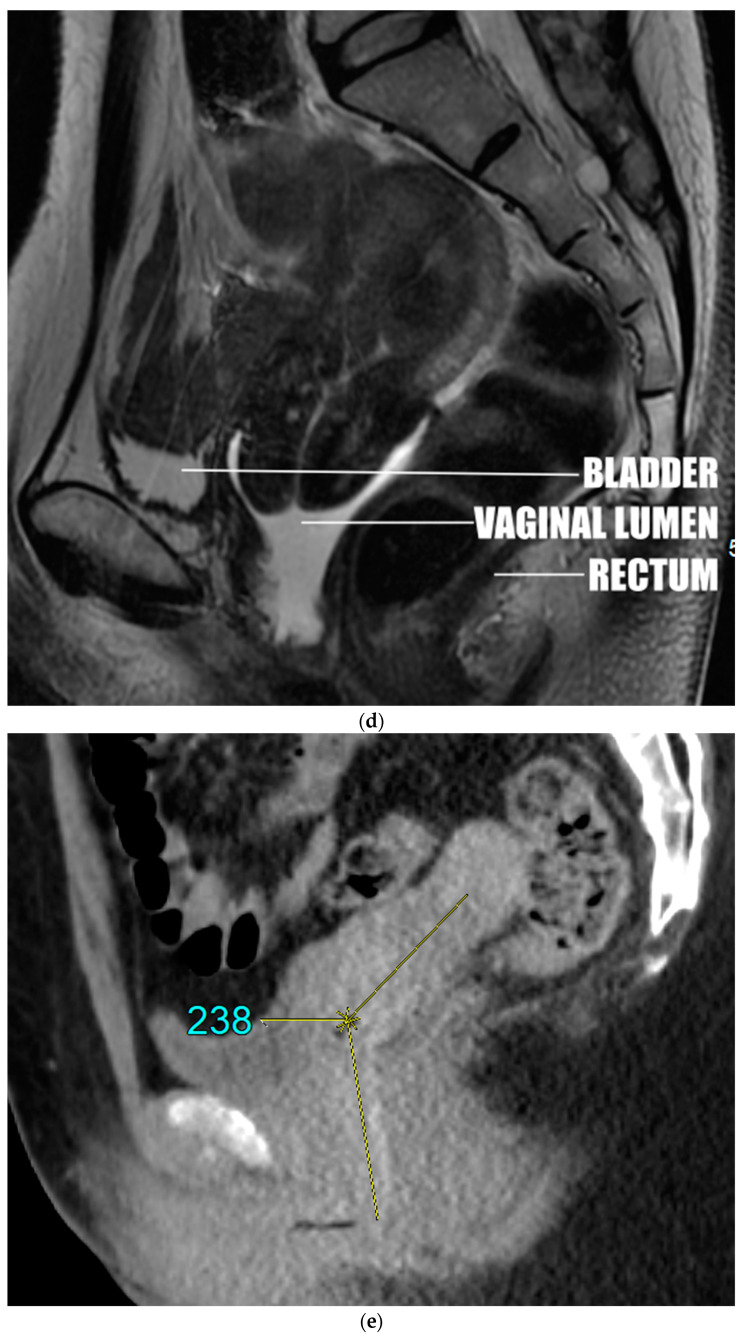
(**a**) Anteverted uterus with the fundus near the pubic body. (**b**) Anteverted uterus in the most common position, close to perpendicular. (**c**) Anteverted uterus, above perpendicular, separated from the bladder. (**d**) Mildly retroverted uterus inserting at the apex of the vagina. (**e**) Markedly retroverted uterus inserting on the posterior wall of the vagina. Sample lines were drawn, and a sample measurement was placed on this image. The double layered line was drawn from the introitus of the vagina to the center of the surface of the cervix. The single layered line was drawn from this point parallel to the axis of the cervix.

The angle of the axis of the cervix with the axis of the vagina was measured on T2 sagittal images of the pelvic MRI scans and the sagittal reconstructions of the pelvic and abdominopelvic CT scans using the measuring tool of the McKesson Radiology Station (12.2.3 64-bit version) in all cases. A line was drawn from the introitus of the vagina to the cervical os or the center of the surface of the cervix if the os could not be visualized. A second line was drawn from this point parallel to the axis of the cervix (see sample measurement lines in Figure 1e). This measurement was subtracted from 180 degrees to obtain the angle from the apex of the vagina. The uterus was considered retroverted if the axis of the cervix was posterior to or the same as the axis of the vagina, or anteverted if the axis of the cervix was anterior to the axis of the vagina. These measurements are consistent with the definitions of uterine anteversion and retroversion used in the imaging [13,15] and anatomic literature [16]. The measurements were entered into Excel (2016) (Microsoft, Seattle, WA, USA).

**Figure 2:**
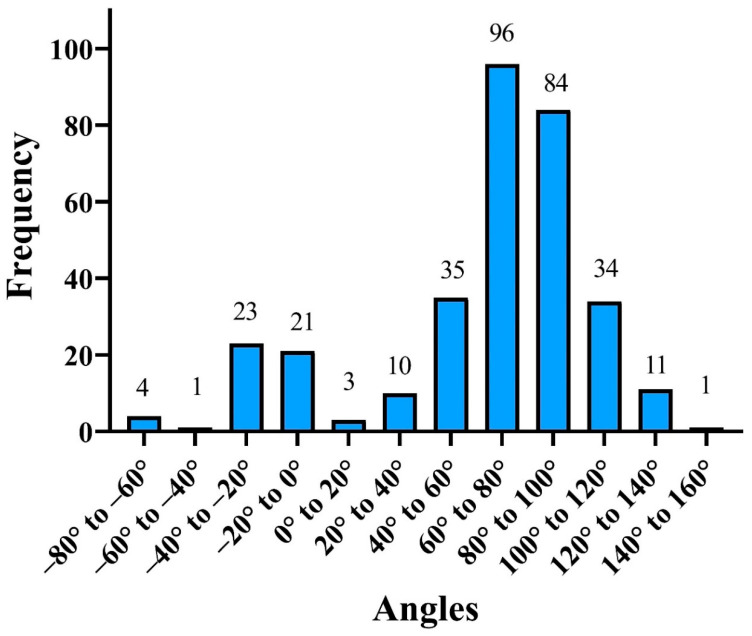
Position of the uterus in 20-degree increments.

Anteverted uteri were assigned positive numbers and retroverted uteri were assigned negative numbers because of our measurement method. The position of the uterus was divided into 20-degree increments and the number and percent of cases in each increment were calculated (Figure 2). Uteri from 40 to −40 degrees off the axis of the vagina were designated as apically inserted. Anteverted uteri with angles above 40 degrees were designated as anteriorly inserted. Retroverted uteri with angles below −40 degrees were designated as posteriorly inserted. The number and percent of anteverted and retroverted uteri with anterior, apical, and posterior insertions by measurement were calculated for the average between the readers, each individual reader, and for CT and MRI scans. The Chi square test was used to compare the proportion of retroverted uteri with apical insertions and the proportion of anteverted uteri with apical insertions for all the above categories. The average angle and standard deviation of the retroverted and anteverted uteri were calculated and compared by a paired *t* test. Test repeatability based on the readings of the two individual readers (or interobserver reliability) was assessed using an intraclass coefficient (ICC) by fitting a two-way random effects model and an absolute agreement. An acceptable level of interobserver reliability was set at an ICC of 0.50, good reliability at 0.75, and excellent reliability at 0.90. The ICC analysis was conducted for all measurements combined as well as for the readings of CT and MRI scans, and the outcomes were expressed as ICC and the respective 95% confidence interval (95%CI). Data analysis was performed in Microsoft Excel (2016) and R software (version 4.1.1) (R Core Team, Auckland, New Zealand).

One reader (A.A.) observed and recorded changes in the anatomic relations of the uterus to surrounding structures as the angle of version (from mild anteversion to marked retroversion) and insertion site (anterior, apical, posterior) changed, as displayed by CT and MRI. This primarily involved changes in the position of the uterus relative to the urinary bladder, vagina, and rectum (Figure 1a–e).

## 3. Results

### 3.1. Scan Categories

In total, 489 scans were reviewed, and 161 scans were excluded because the uterus was absent. Two scans were excluded because the uterus was prolapsed, and three scans were excluded because they were not technically adequate to permit measurement (two secondary to streak artifact from hip prostheses and one secondary to excessive motion artifact). Measurements were performed on 323 scans (228 CT scans, 63 MRI scans with gel distention of the vagina, 32 MRI scans without gel distention). None of the CT and MRI scans were performed on the same patient.

### 3.2. Uterine Positions

Uterine position was distributed along an arc from 145 to −70 degrees (Figure 1a–e). There was a bimodal distribution of uterine position with a large peak at 60 to 100 degrees and a smaller peak at 0 to −40 degrees (Figure 2).

Uterine retroversion was identified on 49 scans (15.2%) by the average measurement of the two readers of all scans included in the study. Forty-four retroverted uteri (89.8%) inserted at the apex of the vagina. The remainder (5, 10.2%) inserted on the posterior wall of the vagina. Uterine anteversion was present in 274 scans (84.8%) by the average measurement of the two readers. Thirteen anteverted uteri (4.7%) inserted at the apex of the vagina. The reminder (261, 95.3%) inserted on the anterior wall. Similar findings were obtained for each individual reader and for CT and MRI scans. The Chi square test demonstrated a statistically significant difference between the frequency of apical insertions in retroverted uteri and apical insertions in anteverted uteri for the average measurement of the two readers (*p* < 0.05), for each individual reader, and for CT and MRI scans (Table 1). Inter-rater agreement was judged as excellent, as indicated by an ICC of 0.976 (95% confidence interval, 0.970 to 0.981) for all measurements included in the study. Similarly, the ICC judges showed excellent agreement for MRI (ICC = 0.994, 95%CI, 0.991 to 0.996) and CT measurements (ICC = 0.966, 95%CI, 0.956 to 0.974).

The mean angle of the anteverted uteri by the average measurement of the two readers was 79.7 degrees (SD 23.1), and the median angle was 80 degrees. The mean angle of the retroverted uteri was −24.4 degrees (SD 24.4), the median angle was −21 degrees. The *t* test demonstrated a statistically significant difference between the mean angles of the anteverted and retroverted uteri (*p* < 0.05).

### 3.3. Anatomic Relations of the Uterus

Observations of CT and MRI images showed that in 236 of 261 cases of anteriorly inserted anteversion, the uterine fundus was superior to and contiguous with the urinary bladder, which is supported by the urethra and the pubic body. The cervix was superior to and contiguous with the vaginal walls and the rectosigmoid colon, which is supported by the pelvic floor (Figure 1a,b).

In the remaining 25 cases of anteriorly inserted anteversion the uterine fundus was superior to and separated from the bladder, but the cervix remained immediately superior to the vaginal walls and rectosigmoid colon (Figure 1c). One uterus considered apically anteverted by measurement had the same anatomic relations.

The remaining 12 apically inserted anteverted uteri were positioned vertically, posterosuperior to the urinary bladder at a shallow angle and anterior to the rectosigmoid. The cervix was surrounded by the vaginal walls and the vaginal lumen was below the cervix. In all 44 cases of apical retroversion, the cervix was positioned vertically, posterior to the bladder and anterosuperior to the rectosigmoid at a shallow angle. The cervix was surrounded by the vaginal walls (Figure 1d). In four of these cases, the uterine fundus was contiguous with the presacral soft tissues or lower lumbar spine. In five of these cases, the uterine fundus was superior to and contiguous with the bladder when the uterus was enlarged and anteflexed.

All five posteriorly inserted retroverted uteri were superior to and contiguous with the rectosigmoid colon, which was supported by the pelvic floor and coccyx (Figure 1e).

### 3.4. Demographic and Clinical Data

Ages of patients ranged from 21 to 96 years, with a mean age of 50.6 years (SD 12.2) and a median age of 52. Abdominal masses and acute abdominal conditions were the most common indications for the CT scans. Outpatient gynecologic conditions were the most common indications for the MRI scans (Table 2).

## 4. Discussion

In this study, we found that the uterus can be located along an arc extending from the pubic bone and urinary bladder to the coccyx and rectum (Figure 1a–e). Of the retroverted uteri, 89.8% were located along an arc of less than 40 degrees past the plane of the vagina before rotation was limited by the rectosigmoid colon, the lower lumbar spine, or the sacrum (Figure 1d), and 10.2% of retroverted uteri rotated further and inserted in the posterior wall of the vagina (Figure 1e). Hence, our study demonstrates that most retroversion is mild with resultant insertion of the cervix into the vaginal apex. This results in alignment of the axis of the cervix with the axis of the vagina.

We found that most anteverted uteri (95.3%) (Figure 1a–c) inserted in the anterior wall of the vagina centered on a peak of 60 to 100 degrees. A much smaller proportion of anteverted uteri (4.7%) than retroverted uteri inserted at the vaginal apex. Hence, a much smaller proportion of anteverted uteri would be subject to the postulated mechanisms leading to uterine prolapse.

Our study shows that the anatomic alignment of the uterus assumed by three proposed mechanisms of the apparent association of uterine prolapse with uterine retroversion does occur. The presence of this anatomic alignment suggests that these mechanisms take place, which in turn suggests that uterine retroversion is a causative factor for uterine prolapse. The “pistoning” mechanism proposes that damage to multiple pelvic fascial supports results in prolapse [1,5]. The “twisting” mechanism proposes that damage to the transverse cervical ligaments secondary to damage to the uterosacral ligaments results in uterine prolapse [8]. The “widened urogenital hiatus” mechanism proposes that damage to the levator ani combined with alignment of the uterus with the vagina secondary to retroversion results in uterine prolapse [6,7]. Hence, damage to the uterosacral ligament, transverse cervical ligament, and the levator ani have all been proposed as factors in uterine prolapse [6,7,8]. Damage to the round ligament may also play a role as it is thought to hold the uterus in anteversion [17]. Retroversion causing damage to endopelvic fascial supporting structures, damage to fascial supporting structures causing retroversion, and damage to fascial supporting structures causing retroversion leading to further damage to fascial supporting structures have all been proposed as causes for uterine prolapse [1,5,6,7,8]. Longitudinal studies of women with uterine retroversion, evaluating the timing of uterine prolapse relative to the timing of uterine retroversion and the timing of events which could cause pelvic floor damage, could help determine which, if any, or if all, of these mechanisms are involved. Studies utilizing imaging of the endopelvic supporting structures of the uterus may provide further information on the mechanisms of uterine prolapse. Existing studies utilizing imaging of the endopelvic supporting structures of the uterus provide information on the mechanisms of uterine support [18,19].

Observations of images from this study appear to show that most anteriorly inserted anteverted uteri receive support from the urinary bladder, the bodies of the pubic bones, the anterior and posterior walls of the vagina, the rectosigmoid colon, and the pelvic floor (Figure 1a). The only structures below apically inserted uteri are the vaginal lumen and the urinary bladder or rectosigmoid colon at shallow oblique angles (Figure 1c). This suggests that there is less support from surrounding organs for apically inserted uteri, providing an additional possible explanation for the greater incidence of uterine prolapse in uterine retroversion.

There is evidence of an association of uterine prolapse and other pelvic floor malfunctions with uterine retroversion. In an observational study performed in Nepal published in 2018 on 120 consecutive women attending a general gynecology clinic for a wide spectrum of symptoms, 68 subjects (60%) had uterine retroversion, 45 (38%) had significant prolapse, 25 (21%) had a significant cystocele, and 10 (8%) had a significant rectocele under the International Continence Society Pelvic Organ Prolapse Quantification System. There was a statistically significant association (*p* 0.062) of uterine retroversion with uterine prolapse [2]. In an observational study performed in Australia published in 2005 of 592 women presenting for initial urogynecological assessment, including urodynamic testing, grade 2–4 uterine prolapse was 4.5 times more prevalent in retroversion than anteversion, the prevalence of grade 2–3 cystocele was 1.9 times greater in retroversion than anteversion, and enterocele was 4.7 times greater in retroversion than anteversion [1]. In an observational study performed in India published in 1948, 52% of women diagnosed with retroversion after a difficult vaginal delivery had symptoms of prolapse [3]. In an observational study performed in Canada published in 1941 of 206 women with uterine retroversion who had been admitted to hospital for a spectrum of gynecologic disorders, there were 24 subjects with uterine prolapse and 32 subjects with cystoceles or rectoceles [4]. In addition, Mayo and another author have made anecdotal references to a universal or near universal association of uterine retroversion with uterine prolapse [7,20]. Although the reproducibility of this association suggests that it is a true association, well-controlled longitudinal studies following subjects with retroversion for the development of uterine prolapse would be necessary to prove the association. As the prevalence of pelvic floor dysfunction is high [21] and expected to increase [22], such studies can be justified. In the authors’ experience, many institutions routinely determine and record the version of the uterus when performing pelvic ultrasound and pelvic MRI. The records from these scans could be used to identify subjects for such a study.

A study using MRI to compare uterine version in women with prolapsed uteri to uterine version in women without prolapse (among other comparisons between the two groups) found a statistically significant increase (*p* < 0.001) in posterior rotation of the uterus in women with prolapse compared to women without prolapse. The study also found that “the uterus is retroverted…in patients with pelvic organ prolapse” [12]. A similar study found a statistically significant increase (*p* < 0.05) in posterior rotation of the uterus in women with prolapse compared to women without prolapse [23]. These studies provide some additional support for an association of uterine prolapse with uterine retroversion but do not establish that prolapse was caused by retroversion. Retroversion could be caused by prolapse or retroversion and prolapse could have the same underlying cause.

This study does have drawbacks. The scans were all performed with the subject in supine position. The uterus is a mobile organ so the position of the uterus could change in the upright position. However, it appears likely that the limitation of posterior rotation of the uterus by the rectosigmoid colon or sacrum would persist when the patient is upright in most cases, so the alignment of the axis of the uterus with the axis of the vagina would likely be maintained in most cases. A study of uterine position with the subjects in the upright position could confirm or exclude this. Conceivably, gel distention of the vagina could affect uterine position. However, as the measurements of uterine angles in CT scans, where gel distention was never used, were similar to the measurements of uterine angles in MRI scans, where gel distention was used in most cases, this does not appear likely. The study was performed by retrospective review, which can result in patient selection bias. Additionally, obstetric history was not readily available for most of the women in this study, so this aspect of the patient population cannot be assessed. However, this is a study of a possible anatomic precursor of uterine prolapse, not prolapse itself, so any bias in patient selection is unlikely to be critical.

## 5. Conclusions

Our study confirmed that the cervix of most retroverted uteri, as well as mildly anteverted uteri, aligns with the long axis of the vagina. Hence, our study demonstrated that the alignment necessary for the proposed mechanisms of the apparent association of uterine prolapse with uterine retroversion does occur, which increases the tenability of the association. Further, anatomic relations seen on the images from this study suggest that retroversion diminishes the support of the uterus by surrounding structures (Figure 1a–d).

## Figures and Tables

**Table 1:** Frequency distribution of apical retroverted and apical anteverted uteri.

Parameter	Anteverted	Retroverted	*p*
Average measurement	13/274 (4.74%)	44/49 (89.80%)	<0.05
AA measurement	17/274 (6.20%)	44/49 (89.80%)	<0.05
MT measurement	15/274 (5.47%)	43/49 (87.76%)	<0.05
MRI measurement	6/78 (7.69%)	17/17 (100.00%)	<0.05
CT measurement	7/189 (3.70%)	27/32 (84.38%)	<0.05

**Table 2:** Clinical indications for CT and MRI scans.

Indications for Imaging	Frequency
**CT INDICATIONS**	
Abdominal pain	72
Abdominal mass	27
Hematuria	22
Metastasis	16
Elevated white cell count/Infection	11
Pelvic pain	8
Abscess	6
Hernia	6
Adnexal cyst/mass	5
Pancreatitis	4
Abdominal aortic aneurysm	4
Trauma	4
Abdominal mass	3
Hepatic steatosis/hepatitis	3
Pancreatic cyst/mass	3
Miscellaneous	34
**MRI INDICATIONS**	
Adnexal cyst/mass	30
Uterine mass	20
Abnormal uterine bleeding	11
Pelvic pain	11
Elevated white cell count/infection	4
Endometriosis	4
Polycystic ovary	3
Miscellaneous	14

## Data Availability

The data presented in this study are available upon request from the corresponding author if the Memphis VAMC IRB approves the request. The data are not publicly available due to privacy regulations of the Department of Veterans Affairs.
